# One-pot synthesis of a microporous organosilica-coated cisplatin nanoplatform for HIF-1-targeted combination cancer therapy

**DOI:** 10.7150/thno.41077

**Published:** 2020-02-03

**Authors:** Xiaojuan Zhang, Chuanchuan He, Xiaoguang Liu, Yan Chen, Pengxuan Zhao, Chen Chen, Ruicong Yan, Minsi Li, Ting Fan, Bouhari Altine, Tan Yang, Yao Lu, Robert J. Lee, Yongkang Gai, Guangya Xiang

**Affiliations:** 1 School of Pharmacy, Tongji Medical College, Huazhong University of Science and Technology, Wuhan 430030, China.; 2 Department of Nuclear Medicine, Union Hospital, Tongji Medical College, Huazhong University of Science and Technology, Wuhan 430022, China; 3 Hubei Province Key Laboratory of Molecular Imaging, Wuhan 430022, China; 4 Division of Pharmaceutics and Pharmacology, The Ohio State University, Columbus, OH 43210, USA

**Keywords:** cisplatin, nanoparticle, HIF-1, acriflavine, combination therapy

## Abstract

Nanoparticle formulations have proven effective for cisplatin delivery. However, the development of a versatile nanoplatform for cisplatin-based combination cancer therapies still remains a great challenge.

**Methods**: In this study, we developed a one-pot synthesis method for a microporous organosilica shell-coated cisplatin nanoplatform using a reverse microemulsion method, and explored its application in co-delivering acriflavine (ACF) for inhibiting hypoxia-inducible factor-1 (HIF-1).

**Results**: The resulting nanoparticles were tunable, and they could be optimized to a monodisperse population of particles in the desired size range (40-50 nm). In addition, organic mPEG2000-silane and tetrasulfide bond-bridged organosilica were integrated into the surface and silica matrix of nanoparticles for prolonged blood circulation and tumor-selective glutathione-responsive degradation, respectively. After reaching the tumor sites, cisplatin induced cancer cell death and activated HIF-1 pathways, resulting in acquired drug resistance and tumor metastasis. To address this issue, ACF was co-loaded with cisplatin to prevent the formation of HIF-1α/β dimers and suppress HIF-1 function. Hence, the efficacy of cisplatin was improved, and cancer metastasis was inhibited.

**Conclusion**: Both *in vitro* and *in vivo* results suggested that this core-shell nanostructured cisplatin delivery system represented a highly efficacious and promising nanoplatform for the synergistic delivery of combination therapies involving cisplatin.

## Introduction

In recent decades, cisplatin (cis-diamminedichloroplatinum II, or DDP), a classic platinum-based antitumor agent that exerts significant genotoxicity, has been extensively applied in clinical treatment and laboratory research 1. Because of its broad spectrum of anticancer activity, cisplatin is used to treat various solid tumors such as lung, liver, and ovarian cancers 2. However, serious side effects such as myelosuppression, acute nephrotoxicity, and chronic neurotoxicity might occur during cisplatin chemotherapy because of its nonselective tissue distribution 3. Fortunately, nanoparticle (NP) formulations can improve drug accumulation in tumor tissues via enhanced permeability and retention (EPR) effects, thereby reducing adverse side effects 4. To date, a series of nanocarriers including liposomes, polymeric micelles, dendrimers, and inorganic NPs such as calcium phosphate NPs, gold NPs, and mesoporous silica NPs have been investigated for cisplatin delivery 5,6.

Cancer cell resistance is also an important issue in cisplatin chemotherapy 7,8. Hypoxia-inducible factor-1 (HIF-1), the central transcription factor in hypoxic tumor cells, plays a vital role in tumor cell resistance to various cancer therapies including radiation therapy, photodynamic therapy, and chemotherapy 9. It was reported that multidrug resistance protein-2 (MRP2), which is associated with cisplatin efflux, was regulated by HIF-1 10. In addition, HIF-1 increases the expression of glutathione (GSH), a small peptide that can bind with some heavy metal ions, including platinum (II) 11. Thus, resistance to cisplatin is highly associated with HIF-1 expression. It is worth noting that HIF-1 pathways can be activated even in normoxic cells during cancer treatment. Prior research found that exposure of cancer cells to chemotherapeutic drugs induced HIF-1α stabilization and activation in a reactive oxygen species (ROS)-dependent manner 12,13. Moreover, other therapies such as photodynamic therapy, ultrasound therapy, and nutrient starvation therapy could also exacerbate HIF-1 signaling via O_2_ consumption 14,15. In response to cisplatin, cells generate ROS through nicotinamide adenine dinucleotide phosphate oxidase-mediated O_2_ transformation to H_2_O_2_, resulting in lower O_2_ concentrations and higher ROS levels 16. Thus, it can be envisaged that cisplatin can induce HIF-1 function even under normoxic conditions, and this was confirmed in A549 lung cancer cells in the current study. Therefore, it might be possible to circumvent drug resistance by inhibiting HIF-1 pathways during cisplatin treatment. In this regard, co-delivery of HIF-1 inhibitors with cisplatin might be an effective therapeutic strategy for cancer.

As a potent HIF-1 functional inhibitor, acriflavine (ACF), which was approved as an antibacterial agent by the FDA 17, can bind to HIF-1α and prevent HIF-1α/β dimerization. However, ACF is a hydrophilic and cationic molecule that exhibits a short plasma half-life, with 90% of ACF eliminated within 5 min after intravenous injection 18. Because of its poor tumor accumulation and rapid clearance, tumor growth was only mildly suppressed by a large dose of ACF 17. Therefore, integrating ACF into NPs to improve its pharmacokinetic characteristics is reasonable for ACF-mediated HIF-1 inhibition. Although ACF-delivered NPs have been developed to enhance the effects of different types of cancer therapies including sonodynamic therapy, photodynamic therapy, and immunotherapy 14,19,20, there has been limited research on the ACF-mediated sensitization of chemotherapy using NPs.

**Scheme 1:**
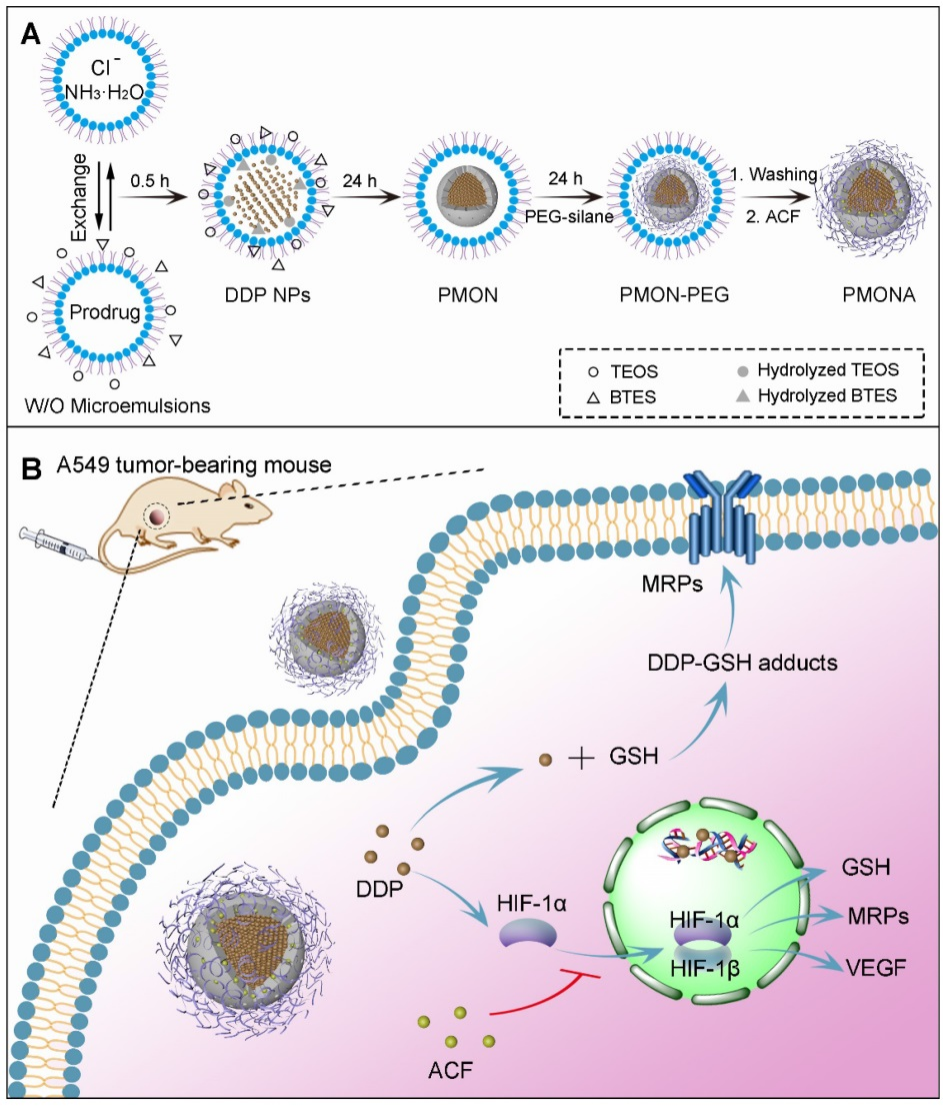
(**A**) Synthetic procedure of PMONA. (**B**) The synergistic anti-cancer mechanism of cisplatin and acriflavine co-loaded in PMONA.

In this study, we developed a facile strategy to synthesize a new type of microporous silica shell-coated cisplatin NP with a core-shell structure and tunable core/shell size and explore its application in synergistic anticancer therapy when loaded with ACF (Scheme 1A). The cisplatin core was synthesized via the precipitation of the hydrophilic cisplatin prodrug (cis-[Pt(NH_3_)_2_(H_2_O)_2_](NO_3_)_2_) and Cl^-^, and silica was produced using a sol-gel process under an alkaline condition. By combining these two reactions in a reverse microemulsion environment, cisplatin-based nanosilica could be synthesized in one pot and functionalized with polymeric mPEG-silane to keep the NPs stealthy 21. To achieve effective drug release in tumor cells, tetrasulfide bond-bridged organosilica was integrated into the NPs for tumor-specific GSH responsiveness 22. Finally, the cationic ACF molecules were loaded into the negatively charged microporous silica shell via electrostatic adsorption. After internalization of the NPs by cancer cells, the outer organosilica shell was degraded by intracellular GSH, leading to NP disintegration and drug release. We hypothesized that the synergistic antitumor effect of the two drugs was based on the following proposed mechanisms (Scheme 1B): (1) cisplatin triggered the transformation of O_2_ into H_2_O_2_, resulting in HIF-1 activation; (2) HIF-1 activated the glutamate-cysteine ligase modifier subunit (GCLM, a GSH synthesis-associated protein) and MRP2, leading to increased GSH-cisplatin binding and drug efflux; (3) HIF-1 increased vascular endothelial growth factor (VEGF) expressed and promoted cancer cell metastasis; (4) ACF inhibited HIF-1 function by binding to HIF-1α, thereby reducing the expression of HIF-1-regulated proteins including MRP2, GCLM, and VEGF; and (5) finally, acquired resistance to cisplatin was reversed and cancer metastasis was inhibited. The enhanced anticancer efficacy was demonstrated both *in vitro* and *in vivo*. In this work, we both highlighted the synergistic antitumor mechanism between cisplatin and an HIF-1 inhibitor to combat acquired resistance and provided a flexible and inducible delivery nanoplatform for cisplatin-based combination therapy.

## Results and Discussion

### Synthesis and optimization of cisplatin microporous silica NPs (PMSN)

First, a hydrophilic cisplatin prodrug, cis-[Pt(NH_3_)_2_(H_2_O)_2_](NO_3_)_2_, was synthesized and concentrated to 200 mM as reported previously 23. Subsequently, the one-pot synthesis of silica shell-coated cisplatin-loaded NPs was conducted using a reverse microemulsion method. As depicted in Scheme 1A, aqueous cisplatin prodrug was dispersed in cyclohexane with the assistance of surfactants to form a transparent and homogeneous water-in-oil emulsion, and silica precursors such as tetraethoxysilane (TEOS) were also added into the system 24. Another well-dispersed emulsion containing Cl^-^ and NH_3_·H_2_O in water phase was prepared similarly. The reactions were started by mixing the two emulsions. Because the precipitation between cis-[Pt(NH_3_)_2_(H_2_O)_2_] (NO_3_)_2_ and Cl^-^ was faster than the sol-gel process, cisplatin cores were formed prior to silica condensation, resulting in distinct cisplatin-rich core/silica shell-structured NPs instead of homogeneous cisplatin/silica hybrid NPs. Notably, it has been demonstrated that most coated inorganic NPs such as Fe_3_O_4_@MSN, ZnO@SiO_2_, and CaCO_3_@SiO_2_ were prepared using stabilizers such as hexadecyltrimethylammonium bromide (CTAB), poly(vinylpyrrolidone) (PVP), or citrate 25-27. This stabilizer-free silica coating strategy may be ascribed to the high affinity between cationic cisplatin cores and the negatively charged Si-OH bond of silica.

**Figure 1:**
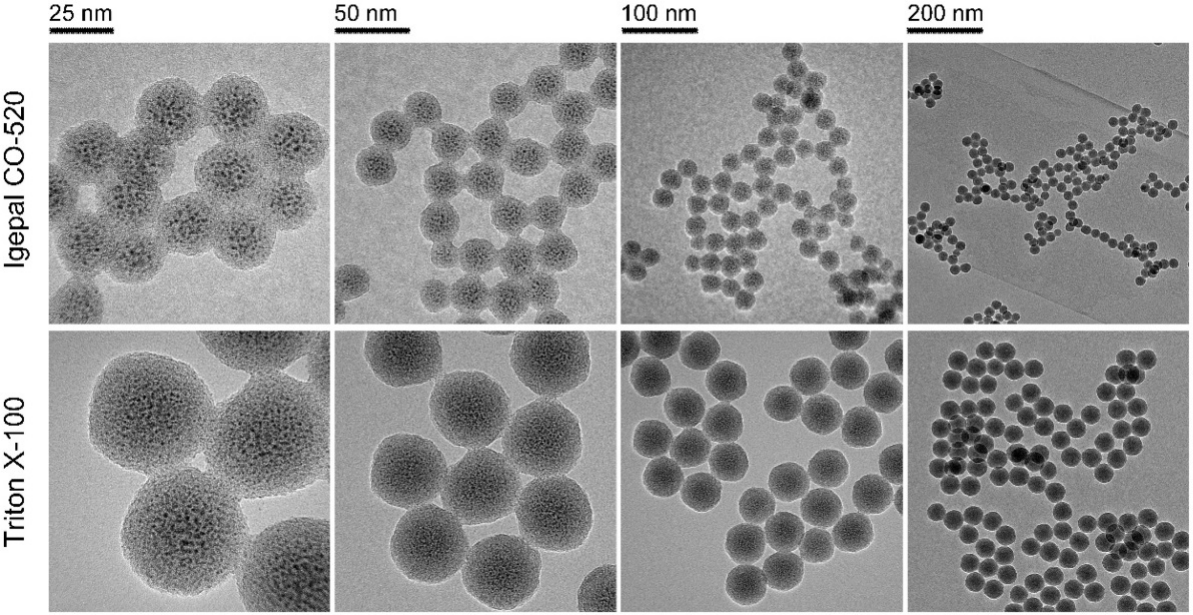
FTEM images of PMSN prepared using different surfactant systems: Igepal CO-520 system (Igepal CO-520: cyclohexane = 30:70 (v/v)) and Triton X-100 system (Triton X-100: Hexanol: cyclohexane = 15:10:75 (v/v/v)).

To obtain an optimized particle size, it is necessary to have insight into the roles of the various synthetic parameters. First, Triton X-100 and Igepal CO-520, two classic surfactants, were selected and assessed. Obviously, both the particle and cisplatin core sizes obtained using Triton X-100 were larger than those obtained using Igepal CO-520 (Figure 1). It is well established that Igepal CO-520 has greater ability to decrease surface tension, and smaller water drops in microemulsion can be formed compared with the Triton X-100 system. As a result, NPs prepared in reverse microemulsions tend to be smaller in Igepal CO-520 systems 28. Second, the effect of the water-to-surfactant molar ratio (R) in NP preparations was also explored. As shown in Figure S1, the synthetic NPs exhibited slightly larger diameters when R was increased in the Igepal CO-520 system, whereas the NPs were smallest and most uniform at R=10 in the Triton X-100 system (Figure S2), which might be attributable to the intrinsic properties of two surfactants.

In addition, the feeding ratio of precursors could affect synthetic NPs. In this experiment, we chose Triton X-100 as a constant surfactant and varied the amounts of cisplatin prodrug and TEOS. As revealed in Figures S3 and S4, a slight difference in particle sizes was observed. Intriguingly, the ratio of core size to shell thickness was correlated with the content ratio of cisplatin prodrug and TEOS. Compared with Igepal CO-520, Triton X-100 is a relatively commercial available chemical and capable of preparing monodisperse NPs with a desired diameter (40-50 nm), which is suitable for EPR effects and tumor penetration 29. Therefore, Triton X-100 was chosen as the surfactant and R was kept at 10 in the subsequent experiments. The concentration of cisplatin prodrug and amount of TEOS were kept at 200 mM and 80 μL, respectively.

### Synthesis and characterization of cisplatin microporous organosilica NPs (PMON)

It has been well established that the organic tetrasulfide bond is redox-sensitive, and it has been widely explored as a biodegradable linker to realize on-demand drug release 30. In this study, bis[3-(triethoxysilyl)propyl] tetrasulfide (BTES), a typical bissilylated organosilica precursor with thioether-bridged groups, was utilized to co-hydrolyze and co-condense TEOS in a microemulsion. After the formation of an organo-bridged silica shell, PEGylation was induced to improve the water solubility and colloidal stability of NPs by adding mPEG2000-silane 31.

**Figure 2:**
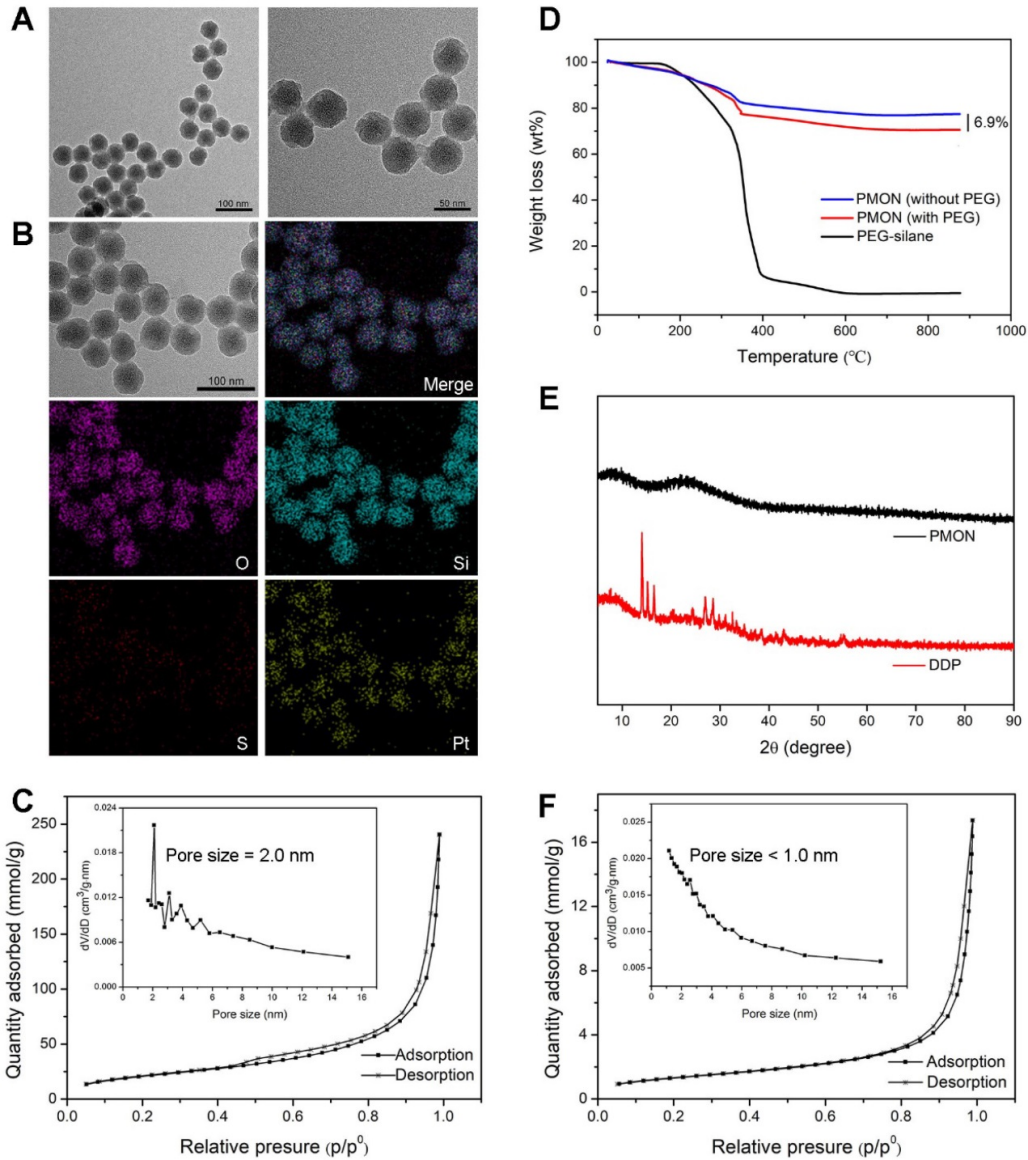
Characterization of PMONA. (**A**) FTEM image of PMON. (**B**) Elemental mapping of PMON. (**C**) The N_2_ adsorption-desorption isotherm and corresponding pore size distribution (inset) of PMON. (**D**) PEG content of PMON assessed by TGA. (**E**) XRD patterns of free DDP and PMON. (**F**) The N_2_ adsorption-desorption isotherm and corresponding pore size distribution (inset) of PMONA.

Similar to the inorganic PMSN, which were prepared without BTES and mPEG-saline, the obtained inorganic/organic hybrid PMON exhibited an average diameter of 45 nm and a shell thickness of 10 nm (Figure 2A). As analyzed via elemental mapping (Figure 2B), the existence of Pt and S signals confirmed the successful encapsulation of cisplatin and integration of tetrasulfide bonds in the NPs. The relative contents of Pt, Si, S, and O were determined using semi-quantitative energy dispersive spectrometry (Figure S5), which further demonstrated the composition of NPs. To assess the drug-loading capacity of the NPs, the surface area and pore size were determined via Brunauer-Emmett-Teller (BET) analysis as 106.8 m^2^·g^-1^ and 2.04 nm, respectively (Figure 2C), indicating the mesoporous structure of PMON, which was helpful for the subsequent loading of small molecules. Furthermore, the amount of PEG grafted onto PMON was obtained via thermogravimetric analysis (TGA) as 6.9 wt. % (Figure 2D). The cisplatin in NPs was also analyzed using X-ray diffraction (XRD). As shown in Figure 2E, the diffraction pattern for cisplatin exhibited several sharp peaks at 13.77, 14.89, 16.21, 26.69, and 28.22°. Interestingly, no characteristic peak was observed in the XRD spectrum of PMON, indicating the amorphous state of cisplatin in the NPs.

### ACF loading and GSH-responsive drug release

**Figure 3:**
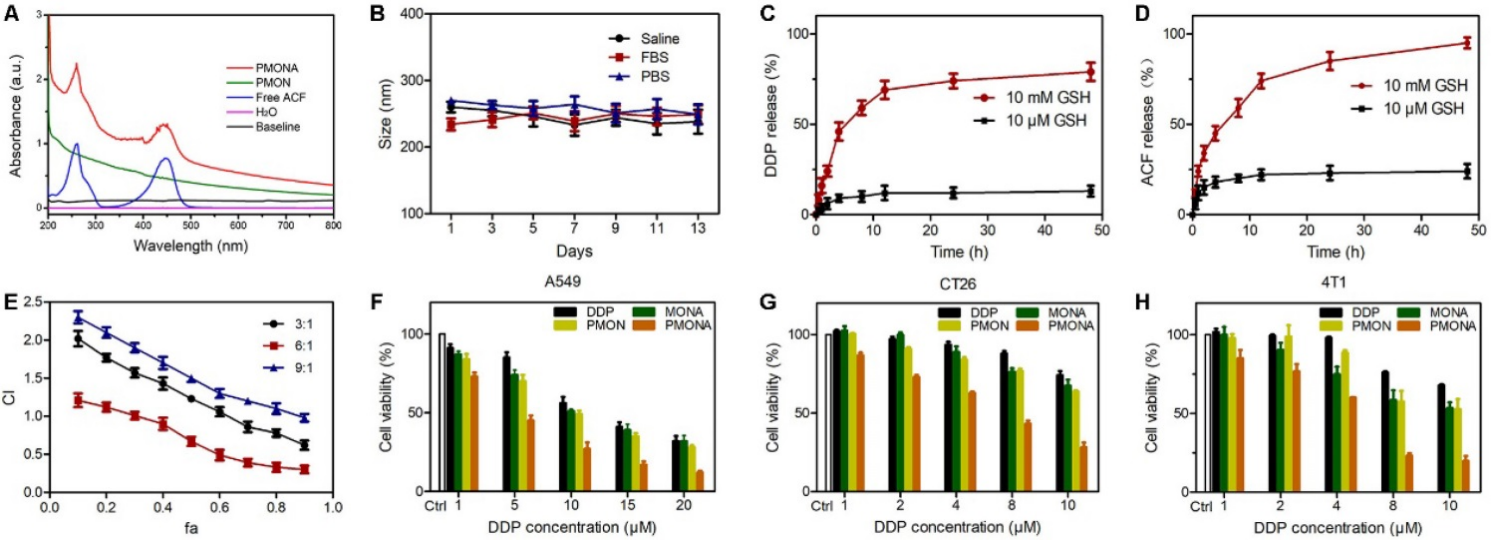
** (A)** UV-vis spectra of the free ACF, PMON and PMONA. **(B)** Time-dependent colloidal stability of PMONA in saline, FBS and PBS at 37 °C. GSH-responsive release of DDP (**C**) and ACF (**D**) from PMONA. (**E**) Combination index (CI) vs. fraction affected (fa) curve after different proportions of DDP and ACF treated on A549 cells for 48 h. Cell viability of A549 cells **(F)**, CT26 cells **(G)** and 4T1 cells **(H)** after different treatments for 48 h.

As mentioned previously, PMON possess a microporous structure and negatively charged surface, which could be suitable for cationic drug loading. ACF molecules are approximately 1.25 nm in diameter, permitting their loading into the inner area of the 2-nm mircopores through electrostatic interactions. By mixing PMON with ACF solution overnight and then washing to remove free ACF, a brown product (PMONA) was obtained after vacuum drying (Figure S6). The hydrodynamic size and zeta potentials of NPs were examined via dynamic light scattering (DLS). As presented in Figure S7, owing to the existence of PEG2000 and the hydration shell, the hydrodynamic size of PMONA was approximately 260 nm, which was substantially larger than that measured via field emission transmission electron microscopy (FTEM). After ACF-loading, the zeta potential of NPs changed from negative to positive, indicating that cationic molecules such as ACF could be encapsulated in this core-shell NPs via electrostatic adsorption (Figure S8). In addition, the pore size of PMONA was less than 1 nm after encapsulating ACF according to Figure 2F, further demonstrating the successful loading of ACF into PMON. As shown in Figure 3A, UV-Vis spectroscopy revealed ACF-represented peaks in the PMONA spectrum, which also proved the successful loading of ACF. To determine the drug-loading capacity of PMONA, inductively coupled plasma atomic absorption spectrometry (ICP-AAS) and UV-Vis spectroscopy were applied to quantify the amounts of loaded cisplatin and ACF, respectively. The drug-loading rates of cisplatin and ACF in PMONA were 8.7% and 3.2%, respectively (see Table S1 in detail).

To evaluate the stability of PMONA, the size changes of NPs in different media were monitored via DLS. No significant size change was observed after incubation PMONA at 37 °C for 14 days (Figure 3B), illustrating its satisfactory biostability. It has been proven that the concentration of intracellular GSH (approximately 0.5-10 mM) is 100-1000-fold higher than that of GSH in extracellular fluids (2-20 μM) 32. To evaluate the GSH responsiveness of PMONA, we performed a drug release study in reductive GSH solutions. As shown in Figure 3C-D, both of the loaded drugs, namely cisplatin and ACF, exhibited faster drug release rates and higher accumulated drug release in 10 mM GSH-containing medium than in 10 μM GSH solution. In addition, the morphology of PMONA after incubation with GSH for 24 h was observed via FTEM (Figure S9), which revealed that the outer organosilica shell was degraded via GSH, leading to NP disintegration. Collectively, we could conclude that the tetrasulfide bond in the silica framework enabled PMONA GSH-responsive degradation and drug release.

### ACF enhances the *in vitro* anticancer efficacy of cisplatin

Because cisplatin is the first-line therapeutic agent for lung cancer, the lung cancer cell line A549 was chosen to evaluate cisplatin-based nanomedicine 33. First, to achieve an optimal combination ratio of these two drugs, MTT assays were performed to evaluate the viability of A549 cells after monotherapy or combination treatment with different molar ratios for 48 h. The synergistic inhibitory effect was assessed using the median-effect method and combination index (CI). CI was plotted as a function of the fraction affected (fa) in A549 cells. CI denotes synergism (CI < 1), additivity (CI = 1), or antagonism (CI > 1). Meanwhile, the fa values represent growth-inhibitory effects 34. As depicted in Figure 3E, when the combination ratio of DDP and ACF was 6:1, a significant synergistic effect appeared at inhibition rates ranging from 30% (fa = 0.3) to 90% (fa = 0.9), indicating that this ratio could realize better anti-tumor synergistic effects than other ratios. Therefore, this ratio was chosen to prepare and evaluate PMONA.

Then the cellular uptake of NPs was carefully evaluated. As illustrated in Figure S10, a larger amount of intracellular Pt was detected in the PMONA group than in the free DDP group after 18 h of incubation. By detecting the green fluorescence of ACF molecules using a fluorescence microscope, we found that after 20 min of incubation, the cellular uptake of ACF in the free ACF group was much greater than that in PMONA group, a finding that was reversed at 60 min (Figure S11). In addition, we confirmed the improved cellular uptake of ACF via flow cytometry (Figure S12). This phenomenon could be ascribed to the different mechanisms in cell uptake, namely the free diffusion of small molecules and endocytosis of NPs. Taken together, these results validated that PMONA could act as an effective nanocarrier with high cellular uptake efficiency.

Further, we investigated the *in vitro* cytotoxicity of NPs in A549 cells. As shown in Figure 3F, the cytotoxic activity of PMON in A549 cells was slightly higher than that of free DDP at the same concentration. As expected, compared with the effects of single drug-loaded PMON and MONA (microporous organosilica NPs loaded with ACF), PMONA exhibited stronger cell cytotoxicity, demonstrating the enhanced synergistic anti-cancer effect of cisplatin and ACF at the cellular level. In addition, these effects were validated using CT26 and 4T1 cells (Figure 3G and 3H).

**Figure 4:**
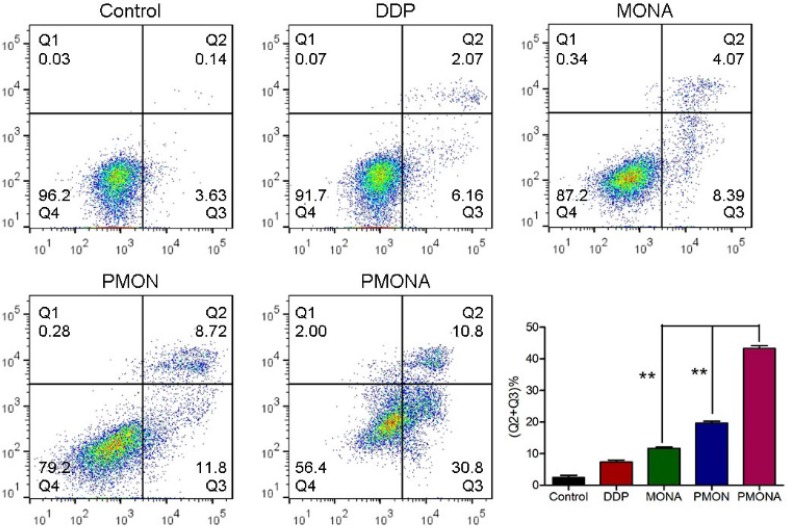
Cell apoptosis of A549 cells following treatments with DDP, MONA, PMON, PMONA at an equivalent concentration of 5 μM cisplatin for 48 h. Untreated cells served as a control. **p < 0.01

**Figure 5:**
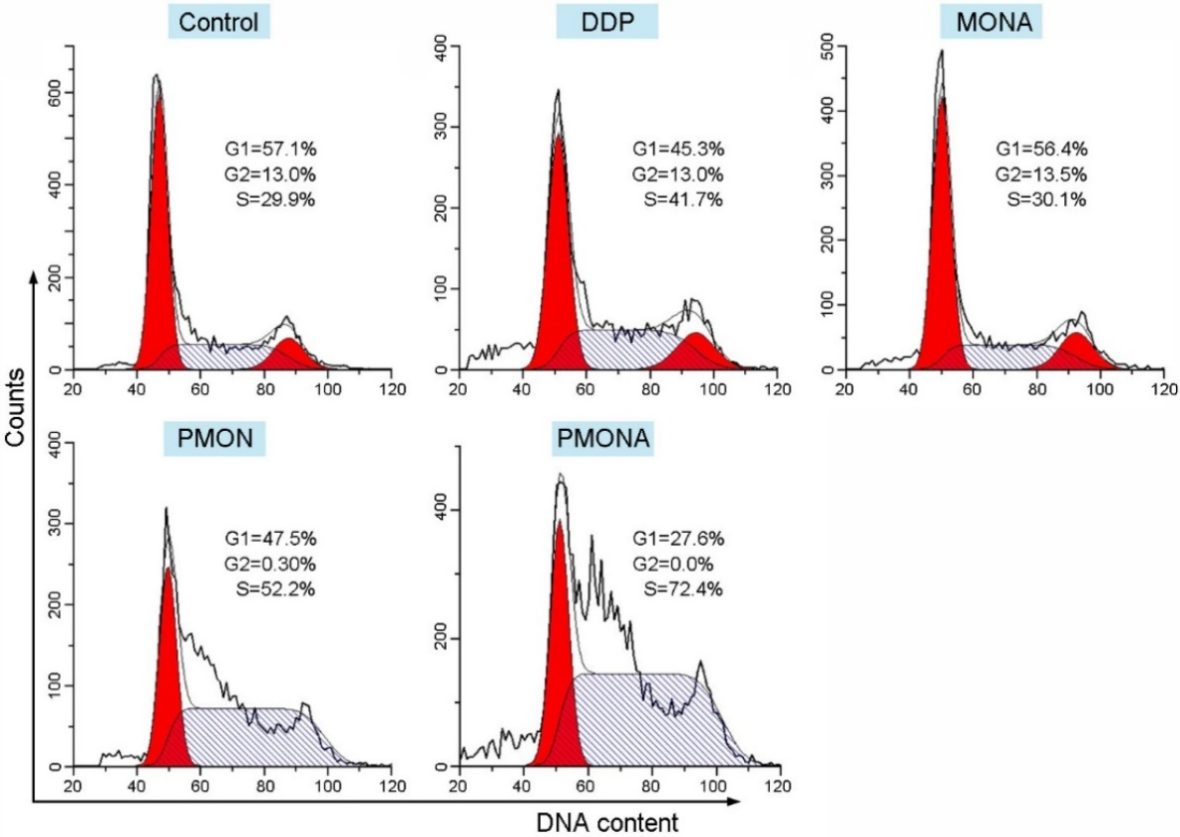
Cell cycle distribution of A549 cells following treatments with DDP, MONA, PMON, or PMONA at an equivalent concentration of 5 μM cisplatin for 48 h. Untreated cells served as a control.

To explore the capacity of PMONA to induce apoptosis in A549 cells, the annexin V-APC/7-AAD method was used to quantitatively analyze apoptosis. As revealed by the results of annexin V-APC/7-AAD double staining, the total percent of apoptotic cells (including early and late apoptotic cells) induced by PMONA reached 41% in A549 cells, far exceeding the findings for PMON and MONA (Figure 4), and demonstrating that the combination of DDP and ACF could bolster cell apoptosis in a co-loaded nanoformulation. Moreover, we evaluated the effect of PMONA on cell cycle progression in A549 cells. As shown in Figure 5, a larger number of cells were arrested in S phase when treated with PMONA, indicating ACF-mediated enhancement of the effects of cisplatin on DNA crosslinking and cell cycle arrest. Taken together, the aforementioned results indicated that co-loaded ACF could improve the *in vitro* chemotherapeutic efficacy of cisplatin.

### Synergistic anticancer mechanism of DDP and ACF co-loaded in PMONA

**Figure 6:**
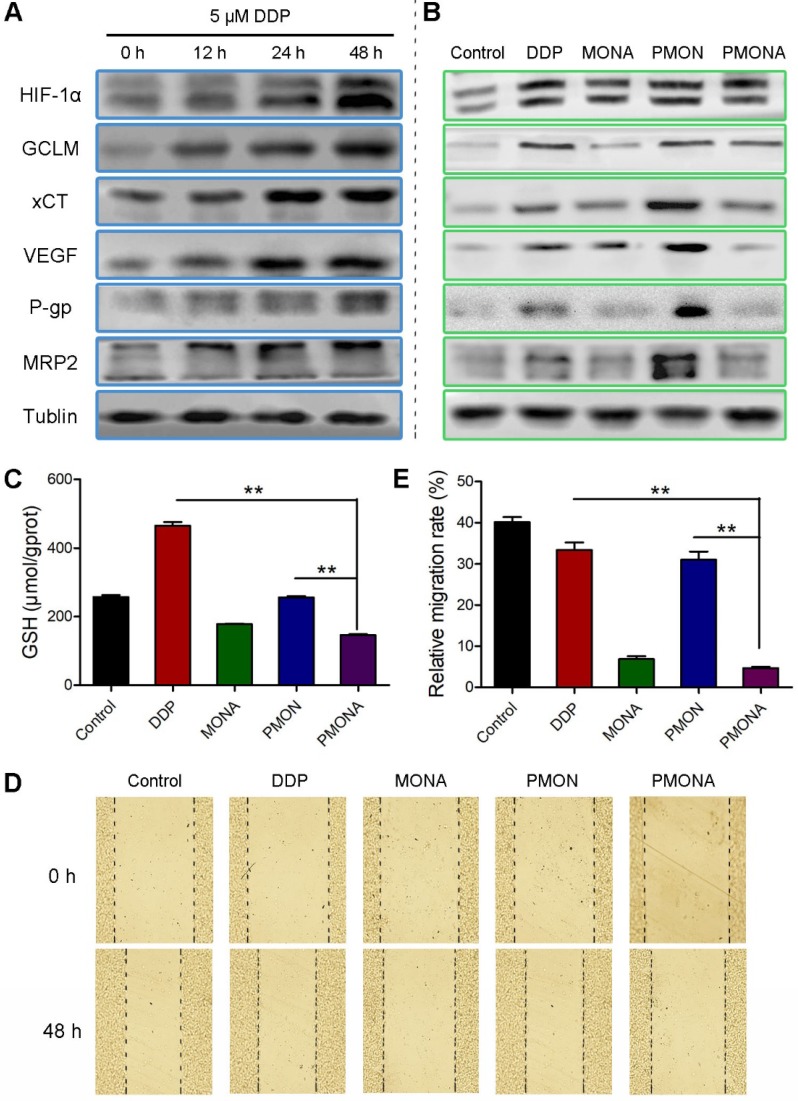
*In vitro* characterization of PMONA. (**A**) the expressions of HIF-1α and the HIF-1-associated proteins after treatments with 5 μM cisplatin for 12 h, 24 h, and 48 h. Effects of DDP, MONA, PMON, PMONA at an equivalent concentration of 5 μM cisplatin on the expression of HIF-1α and the HIF-1-associated proteins (**B**), GSH levels (**C**) and cell migration (**D**, **E**) after 48 h incubation. Untreated cells served as a control. **p < 0.01

Based on the observation that ACF enhanced the anticancer efficacy of cisplatin *in vitro*, the underlying synergistic mechanism was carefully studied. As mentioned previously, chemotherapy could consume O_2_ and produce ROS, further resulting in HIF-1 stabilization. In this study, we verified that A549 cells had an increased tendency to express HIF-1α under prolonged incubation with cisplatin under normoxia. Accordingly, the expression of HIF-1-associated proteins including GCLM, cystine transporter (xCT), MRP2, P-glycoprotein (P-gp), and VEGF was also increased by cisplatin treatment (Figure 6A). Among these, xCT and GCLM are responsible for the synthesis of GSH 35. MRP2 and P-gp can mediate the efflux of GSH-cisplatin adducts, resulting in cell detoxification and cisplatin resistance. VEGF is a well-documented factor responsible for metastasis, that is mainly activated by HIF-1. After incubation with cisplatin/ACF co-delivered using PMONA, all of these cisplatin-induced HIF-1 signals, which were also related to cisplatin resistance and metastasis, were significantly suppressed (Figure 6B). Thus, we concluded that HIF-1 function was inhibited by co-loaded ACF in PMONA, which could explain the synergistic and enhanced anticancer efficacy.

In addition, we determined the effects of NPs on intracellular GSH levels. As expected, PMONA- treated A549 cells displayed obvious lower GSH level than cells treated without ACF (Figure 6C), proving that ACF reduced intracellular GSH level and thereby suppressed GSH binding-associated cisplatin resistance. To verify the inhibition of cancer cell metastasis via ACF-mediated VEGF downregulation, wound-healing assays were performed in A549 cells. As shown in Figure 6D-E, relatively increased migration was observed in cells treated with cisplatin alone. Interestingly, this phenomenon was inhibited by ACF-containing treatments, demonstrating the capacity of ACF co-loaded in PMONA to combat cisplatin-induced cancer metastasis.

### *In vivo* imaging and antitumor studies

**Figure 7:**
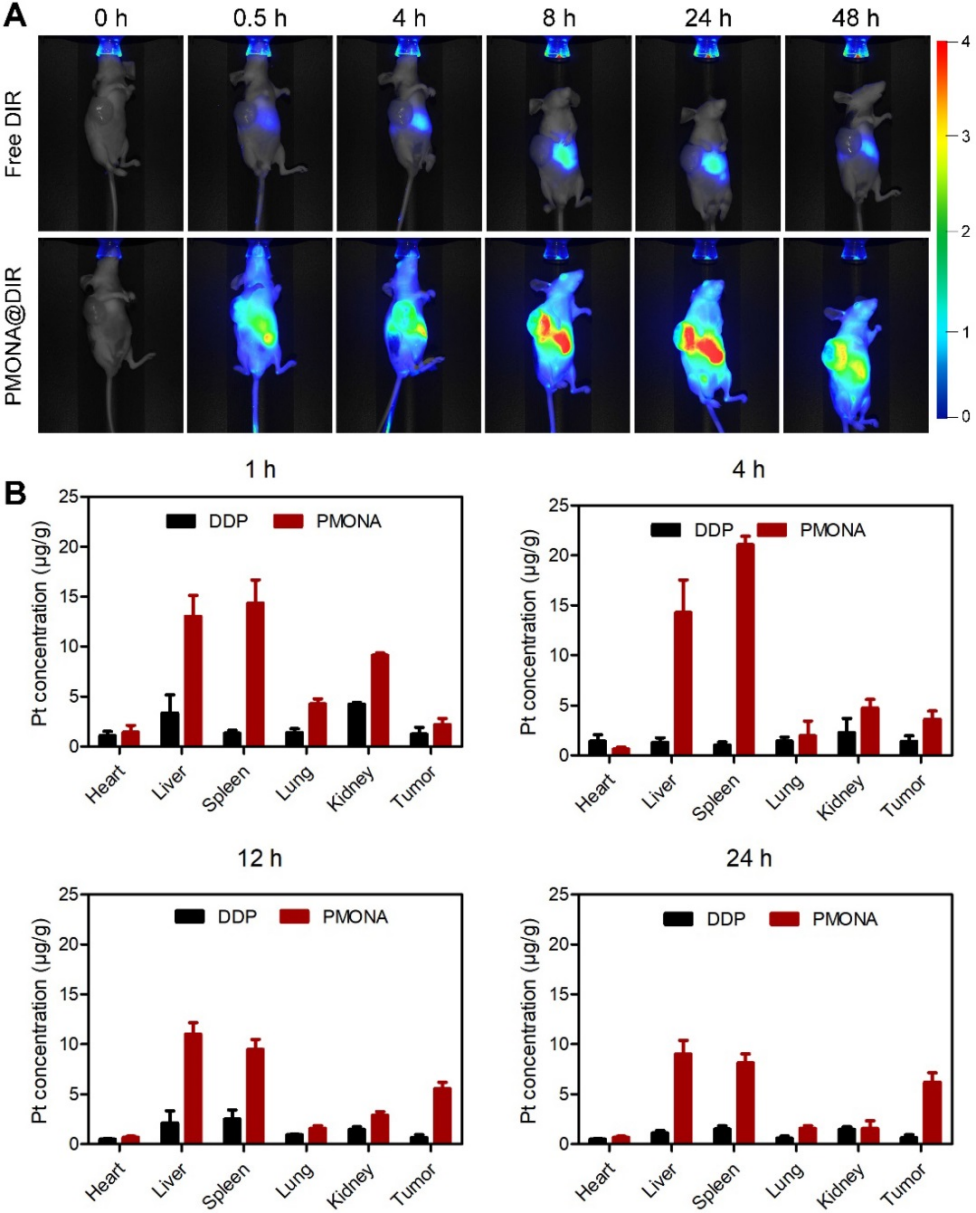
*In vivo* distribution of PMONA. (**A**) Living images of A549 tumor-bearing mice i.v. administrated with free DIR and PMONA@DIR at different time intervals. (**B**) Pt biodistribution in tissues of A549 xenograft tumor-bearing mice at 1, 4, 12 and 24 h after intravenous injection of free DDP and PMONA.

A lung tumor (A549) xenograft model was constructed to investigate the biodistribution of PMONA *in vivo*. As shown in Figure 7A, rapid drug metabolism and clearance from blood circulation were observed in the free DiR group, and a weak fluorescence signal was detected in the liver at 24 h post-injection. However, in the tumor region of PMON@DiR NP-treated mice, the fluorescence signals lasted for more than 48 h, which indicated that PMONA could realize prolonged circulation and efficiently accumulate in the tumor. This could be also concluded by the biodistribution of Pt in tissues at 1, 4, 12 and 24 h after the intravenous injection of free DDP and PMONA (Figure 7B), which was detected in a A549 xenograft tumor-bearing mice model.

**Figure 8:**
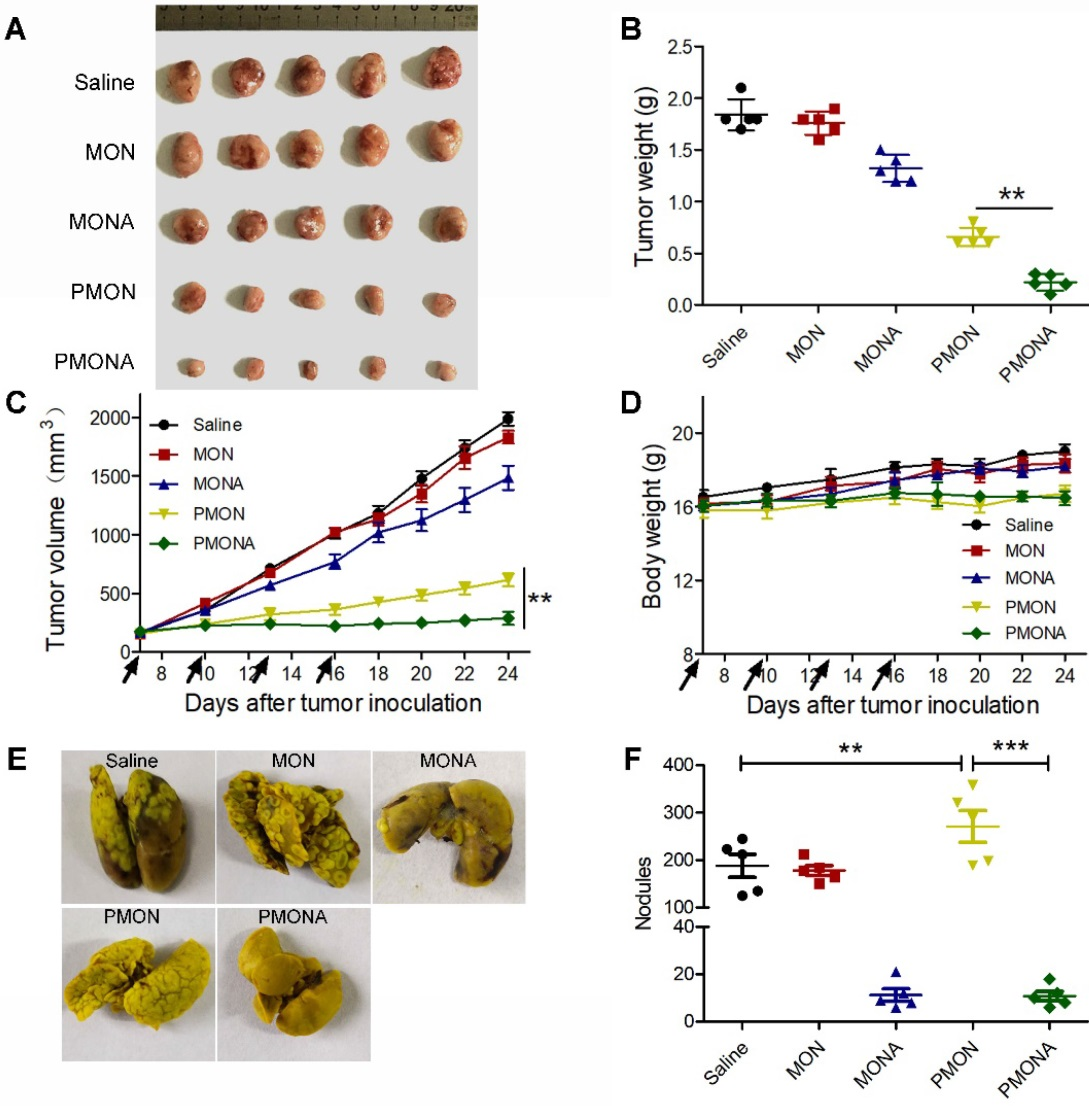
*In vivo* antitumor activities. **(A)** tumor images, **(B)** tumor weight, **(C)** tumor volume and **(D)** body weight. Saline, MON, MONA, PMON and PMONA were injected to A549 tumor-bearing mice through tail vein at an equivalent dose of 2 mg DDP/kg on day 7, 10, 13 and 16 (marked with black arrows). **(E)** Digital photographs of the lung tissues collected at day 20. **(F)** Counting of tumor nodules on the surface of lungs (n = 5). **p < 0.01, **p < 0.001. Saline, MON, MONA, PMON and PMONA were injected to bloodstream metastasis mice model through tail vein at an equivalent dose of 2 mg DDP/kg.

Further, to explore the *in vivo* therapeutic efficacy of PMONA, we started NP treatments when the average volume of A549 xenograft tumors reached approximately 150 mm^3^. From the data shown in Figure 8A-D, dual drug-loaded PMONA exerted stronger anti-cancer effects than NPs loaded with one drug. Meanwhile, no obvious weight loss was observed, indicating the high safety of the nanoformulations. To determine the *in vivo* anti-metastasis effect of PMONA, a bloodstream metastasis model was established via intravenous injection of 4T1 cells. As observed in the images of excised lungs (Figures 8E and S13) and the count of metastatic lung nodules (Figure 8F), ACF-containing nanoformulations obviously inhibited metastasis compared with the effects of saline and cisplatin alone, which proved the anti-metastasis effects of ACF in PMONA.

### *In vivo* biosecurity and antitumor mechanisms

Histological analyses of major normal organs of A549 xenograft tumor-bearing mice including the heart, liver, spleen, lungs, and kidneys confirmed the high biosecurity and limited tissue damage in PMONA-treated mice (Figure S14). In addition, blood biochemistry and hematology analyses of NP-treated mice were conducted to further verify their satisfactory biocompatibility and biosafety (Figure S15).

**Figure 9:**
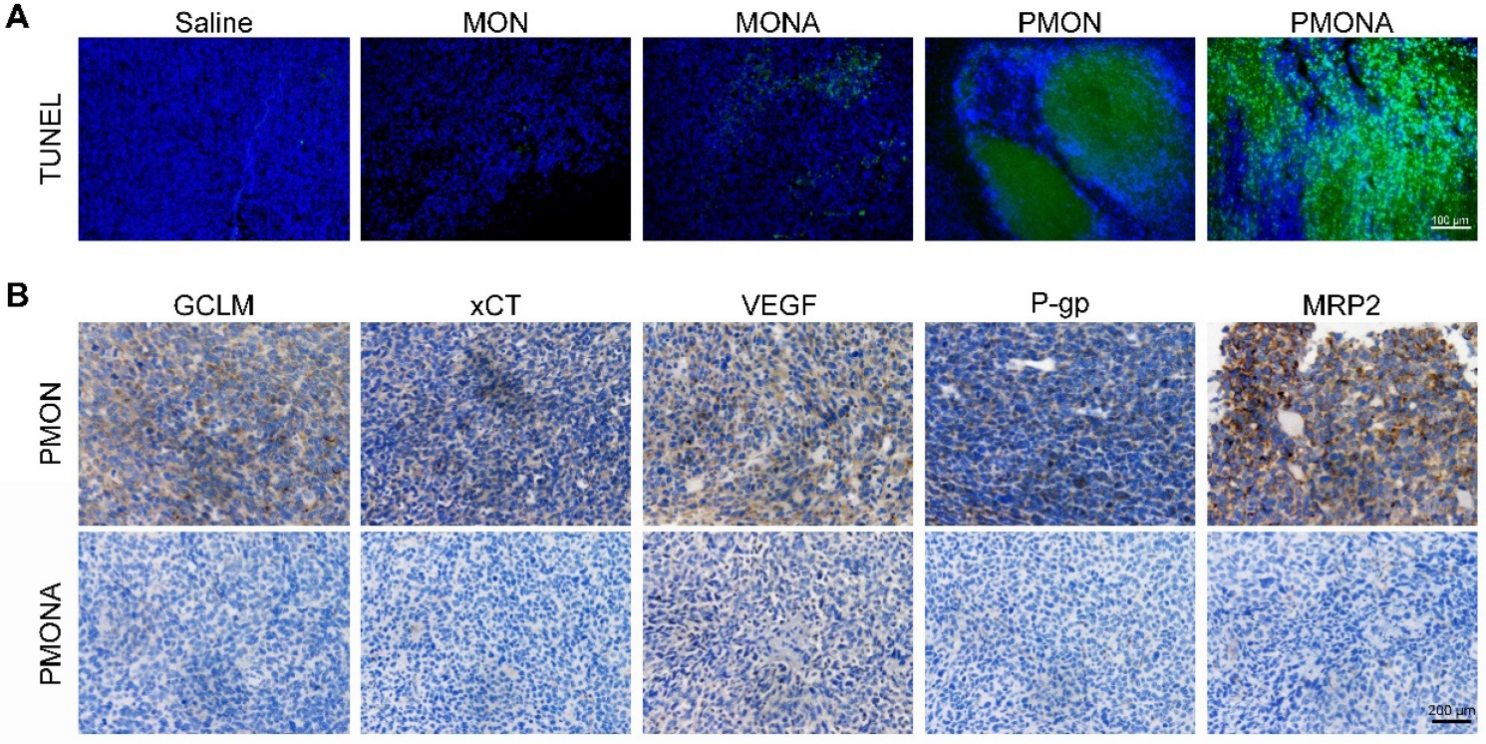
** (A)** Representative images of tumor-site apoptosis determined by TUNEL assay. **(B)** Representative images of immunohistochemical analysis of GCLM, xCT, VEGF, P-gp and MRP2 expression in tumor-site.

As revealed using the TUNEL staining assay (Figure 9A), enhanced apoptosis and cell death were observed in tumor tissues following PMONA treatment. Additionally, tumoral xCT, GCLM, MRP2, P-gp, and VEGF expression was significantly downregulated in the PMONA group compared with the findings in the PMON group (Figure 9B). Collectively, these results verified *in vivo* that ACF could inhibit cisplatin-induced HIF-1 function, consequently enhancing the efficacy of cisplatin chemotherapy and suppressing tumor metastasis *in vivo*.

## Conclusion

For the first time, we developed microporous organosilica-coated cisplatin NPs with core-shell structures and tunable sizes that could be further loaded with small molecules for cisplatin-based combination therapy. The tetrasulfide bridged silicon framework enabled GSH-triggered biodegradation and drug release. To overcome HIF-1-mediated drug resistance and metastasis, ACF was co-loaded into the cisplatin nanoplatform, and an enhanced anticancer effect was achieved both* in vitro* and *in vivo*. We also demonstrated the underlying mechanisms that cisplatin-induced HIF-1 pathways including “xCT and GCLM/GSH/cisplatin binding,” “MRP2 and P-gp/cisplatin efflux,” and “VEGF/cancer metastasis” were successfully inhibited by ACF in the combined nanoformulation. We believe that based on the facile modification and large surface area of porous silica, this core-shell NP can provide a versatile platform for facilitating the development of cisplatin-based combination cancer therapy strategies.

## Supplementary Material

Supplementary materials and methods, figures, and table.

## Abbreviations

DDP: cisplatin
EPR: enhanced permeability and retention effect
HIF-1: hypoxia-inducible factor-1
MRP2: multidrug resistance protein-2
GSH: glutathione
ROS: reactive oxygen species
ACF: acriflavine
GCLM: glutamate-cysteine ligase modifier subunit
VEGF: vascular endothelial growth factor
TEOS: tetraethoxysilane
CTAB: hexadecyltrimethylammonium bromide
PVP: poly(vinylpyrrolidone)
BTES: bis[3-(triethoxysilyl)propyl] tetrasulfide
CI: combination index
xCT: cystine transporter
P-gp: P-glycoprotein
DiR: iodide[1,1-dioctadecyl-3,3,3,3-tetramethylindotricarbocyanine iodide]
