# Supplementary material for: One-pot synthesis of a microporous organosilica-coated cisplatin nanoplatform for HIF-1-targeted combination cancer therapy
Source: Theranostics. 2020 Feb 3;10(7):2918–29. doi: 10.7150/thno.41077 (PMC7053205; doi:10.7150/thno.41077)
Supplement: Supplementary file 1 — Supplementary materials and methods, figures, and table. [file thnov10p2918s1.pdf]

Supporting information

One-pot synthesis of a microporous organosilicon-coated cisplatin nanoplatform for HIF-1–targeted combination cancer therapy

Xiaojuan Zhang[1], Chuanchuan He[1], Xiaoguang Liu[1], Yan Chen[1], Pengxuan Zhao[1], Chen Chen[1], Ruicong Yan[1], Minsi Li[1], Ting Fan[1], Bouhari Altine[2,3], Tan Yang[1], Yao Lu[1], Robert J. Lee[4], Yongkang Gai*[,2,3], Guangya Xiang*[,1]

1. School of Pharmacy, Tongji Medical College, Huazhong University of Science and Technology, Wuhan 430030, China.
2. Department of Nuclear Medicine, Union Hospital, Tongji Medical College, Huazhong University of Science and Technology, Wuhan 430022, China
3. Hubei Province Key Laboratory of Molecular Imaging, Wuhan 430022, China
4. Division of Pharmaceutics and Pharmacology, The Ohio State University, Columbus, OH 43210, USA

Materials and Methods

Materials

Tetrathylorthosilicate (TEOS), bis [3- (triethoxysily) propyl] tetrasulfide (BTES) and Igepal CO-520 were purchased from Sigma-Aldrich Chemical Co. (St. Louis, MO, USA). Triton X-100 was obtained from Aladdin (Shanghai, China). Cisplatin was purchased from the Macklin (Shanghai, China). Fetal bovine serum (FBS) was purchased from Zhejiang Tianhang Biological Technology Co., Ltd. (Hangzhou, China). 3-(4,5-dimethylthiazol-2-yl)-2,5-diphenyltetrazolium bromide (MTT) and 1,10- dioctadecyl-3,3,30,30-tetramethylindotricarbocyanine iodide (DIR) was purchased from Sigma-Aldrich Chemical Co. (St. Louis, MO, USA). Ultrapure Milli-Q water (MW 18.2) was used in all experiments. All chemicals were used as received without further purifications.

Cell culture and animals

The cell lines A549, CT26 and 4T1 was obtained from KeyGEN Biotechnology Co. (Nanjing, China). A549 cells were cultured in DMEM supplemented with 10% FBS, 1% penicillin-streptomycin. The cells were grown in a humidified atmosphere at 37 °C in 5% CO_2. Balb/c and Balb/c nude mice (female, 4-6 weeks old) were obtained from Beijing Huafukang Bioscience Technology Co. (Beijing, China). The experimental protocol was approved by the Committee on Ethical Animal Experiment at Huazhong University of Science and Technology.

Methods

Synthesis of cis- [Pt $(NH_3)_2(H_2O)_2$] $(NO_3)_2$

Cis- [Pt $(NH_3)_2(H_2O)_2$] $(NO_3)_2$ was prepared according to the literature.[1,2] $AgNO_3$ (66.2 mg, 0.39 mmol) was added to 1.0 mL cisplatin suspension (60 mg, 0.20 mmol). The mixture was heated at 60 °C for 3 h and then stirred overnight in a flask kept in dark place. Afterward, the mixture was centrifuged at 12,000 rpm for 10 min to remove the AgCl precipitate. The supernatant was then filtered using a 0.2 μm syringe filter. The concentration of Pt was measured using inductively

coupled plasma atomic absorption spectrometry (ICP-AAS) and adjusted to 200 mM.

Preparation of nanoparticles.

A 4 mL surfactant contained cyclohexane consisting of 200 μL cis- [Pt $(NH_3)_2(H_2O)_2$] $(NO_3)_2$ and 80 μL TEOS was stirred vigorously at room temperature. Another 4 mL mixture consisting of 100 μL KCl (800 mM) and 100 μL $NH_3 \cdot H_2O$ was stirred in a similar manner. After 20 minutes, the two emulsions were mixed and the reaction proceeded for another 24 hours. Then the inorganic cisplatin microporous silica nanoparticles (PMSN) were obtained by the addition of 8 mL ethanol and washed three times with 20 mL ethanol and re-dispersed in 5 mL ethanol. Similarly, for preparing PEGylated biodegradable cisplatin microporous organosilica nanoparticles (PMON), the 80 μL TEOS was replaced by 40 μL TEOS plus 40 μL BTES, and PEG2000-silane was added after 24 hours reaction and allowed to react for another 24 hours, the other procedures were kept the same as in PMSN preparation. Drug-unloaded group microporous organosilica nanoparticles (MON) were synthesized without cisplatin prodrug and KCl.

ACF loading and drug quantification

For ACF loading, an amount of ACF (10 mg/mL, in water) was added to PMON or MON solutions (1 mg/mL in ethanol) and then stirred at room temperature overnight. Then, the excess ACF molecules were removed by ultrafiltration (Millipore) with the molecular weight cut off (MWCO) at 100 kDa to obtain ACF loaded PMON (PMONA) and MON (MONA). The amount of ACF loaded in PMONA or MONA was quantitatively evaluated by UV−vis spectrometry at 450 nm. For the quantification of cisplatin loaded in PMON and PMONA, the samples were digested with concentrated nitric acid and diluted to 2 mL with Milli-Q H_2O, and further subjected to ICP-AAS analysis.

Characterization of nanoparticles

FTEM (Field Emission Transmission Electron Microscopy) samples were prepared by placing a drop of the dispersion onto a copper grid coated with a perforated carbon film, followed by evaporating the solvent at room temperature.

Thermogravimetric analysis (TGA) of the samples was carried out using a Pyris1 thermogravimetric analyzer (PerkinElmer). The TGA curves were recorded in the temperature range of 25−900 °C in air at a heating rate of 10 °C min^{-1}.

The surface area was calculated using the Brunauer−Emmett−Teller (BET) method from nitrogen adsorption–desorption isotherms. The total pore volume was estimated from the adsorbed amounts at a relative pressure (p/p^0) of 0.99. Before measurements, samples were degassed in a vacuum for 6 h at 180ºC.

A UV-vis spectrometer was used to measure the absorption from 200 nm to 800 nm of the solutions (free ACF, PMON and PMONA) in a quartz cell. The hydrodynamic size and zeta potential of nanoparticles were obtained by dynamic light scattering (DLS, Zeta Plus, Brookhaven Instruments, USA) measurement.

***In vitro* drug release**

The release of cisplatin and ACF in PMONA *in vitro* was studied by dynamic dialysis method. PMONA (10 mg) was encapsulated in a dialysis bag (cutoff molecular weight of 2000 Da) and immersed in PBS solution (20 mL) at GSH concentration of 10 μM or 10 mM, which was then put in a shaking table at the shaking speed of 100 rpm at 37 °C. The releasing medium was taken out at a given time for UV-vis analysis and ICP-AAS to determine the ACF-releasing amount and cisplatin-releasing content, respectively. The particles in the dialysis bag were dispersed in ethanol

and observed by FTEM at preset timepoints.

Cellular experiments

The fluorescent of ACF was employed to track the intracellular locations of different formulations. Briefly, the cells were seeded into the 12-well plate at a density of 2 × 10^5 per well. After 24 h, cells were exposed to ACF and PMONA (ACF concentration: 1 μM), respectively. After incubation for different time periods, the initial medium was sucked out and the cells were washed with PBS for three times. Finally, cells were counterstained with 4′, 6-diamidino-2-phenylindole (DAPI: 1.2 μg/mL) for 8 min and then rinsed with PBS. The cells were imaged with a fluorescence microscope (Zeiss LSM 510).

The cytotoxic effects of DDP (free cisplatin), MONA, PMON, PMONA, and free cisplatin and ACF (at the ratio of 6 to 1) were measured using MTT assay. Briefly, cells were seeded into 96-well plates at 5000 cells per well. After 48 h of treatment with different groups, MTT solution (5 mg/mL in PBS) was added and incubated for 4 h (20 μL/well). The supernatants were sucked out carefully and followed by the addition of DMSO. Absorbance was measured using a microplate spectrophotometer (VersaMax, USA).

For apoptosis analysis by FACS, A549 cells (5 × 10^5 per well) were seeded into 6-well plates overnight. Then the cells were incubated with free cisplatin, MONA, PMON, and PMONA for 48 h. The cells were collected and stained using an Annexin V-APC/7-AAD (KeyGEN Biotechnology Co., China) according to the manufacturer's protocol. Stained cells were immediately analyzed on a BD Accuri C6 Flow Cytometer.

After treatment with different drugs, total cellular proteins were obtained using RIPA buffer. Western blotting was performed using standard procedures. The dilution of the primary rabbit antibodies against HIF-1α, GCLM, xCT, VEGF, P-gp, MRP2 and β-tubulin (Cell Signaling Technology, USA) was 1:1000. The secondary antibody (KenGen Biotech, China) was diluted with 2000 times.

The intracellular GSH levels were measured with glutathione assay kit (Solarbio Life Sciences, China). Briefly, cells cultured on a slip of coverglass in 6-well plates were incubated with different groups for 48 h, then total cellular proteins were obtained using RIPA buffer for testing. GSH levels were measured according to the manufacturer's instructions.

To test the anti-migratory potential of PMONA, the A549 cells were seeded in 6-well plates and incubated for 24 h. A perpendicular scratch was generated in the surface of the plate using a pipette tip, followed by extensive washing with PBS to remove cell debris, then incubated with RPMI-1640 medium containing 0.5% FBS and treated with different groups for 48 h. Photographic images were taken from each well at the indicated time after drug treatment, using a light microscope (Zeiss, Germany). The distance that cells migrated through the marked area was determined by measuring the wound width at 48 h after treatment. The experiment was repeated three times.

***In vivo* experiments**

The *in vivo* imaging studies were conducted on A549 bearing mice by a noninvasive optical imaging system (Pearl Trilogy, LI-COR, USA). The fluorescein DIR was used for NPs labeling, which was loaded into the microporous silica shell by simple physical adsorption. Balb/c nude mice were intravenously administrated with PMON@DIR NPs or free DIR at the dosage of 2 μg DIR per mice. After 0.5 h, 4 h, 8 h, 24 h, and 48 h, the fluorescence intensity images of each mouse were recorded by an *in vivo* imaging system.

For the determination of cisplatin concentrations in tissues, A549 xenograft tumor-bearing mice

were given a single dose of free cisplatin (2 mg/kg) and PMONA (2 mg cisplatin/kg), and then sacrificed at 1, 4, 12 and 24 h after the injection. Hearts, livers, spleens, lungs, kidneys and tumor tissues were collected after sacrificed. The concentration of Pt was determined by ICP-AAS as described previously.

To evaluate the *in vivo* tumor inhibition efficacy of NPs, A549 bearing mice were intravenously injected with saline, MON, MONA, PMON and PMONA NPs at 2 mg/kg of DDP or its equivalent on days 7, 10, 13 and 16 (n=6). Tumor sizes and animal body weights were measured every 2 days after treatment. At day 24, a week after the last administration, the mice were sacrificed, then tumors and major organs (heart, liver, spleen, lung, and kidney) were harvested and sectioned into slices for H&E or TUNEL staining analysis.

To establish the metastasis model, female Balb/c mice were given tail vein injection of 4T1 cells suspension (10^6 cells in 0.15 mL saline). The mice were randomly divided into five groups (n = 5) and treated with saline, MON, MONA, PMON and PMONA (2 mg/kg of DDP) on days 2, 5, 8 and 11. Three weeks later, mice were euthanized. The lungs were excised, fixed with Bouin's solution and photographed. The metastatic lung nodules were observed and counted under a magnifying glass.

Statistical Analysis

All data were taken from three independent experiments and then expressed as means ± standard deviation (SD). One-way ANOVA was performed to compare the statistical analysis by Graph Pad Prism 5.0 (San Diego, CA, USA).

Table S1. Characterization of PMONA NPs

	Encapsulation efficiency (%)	Drug-loading content (%)
DDP	56.4±3.1	8.6±1.7
ACF	18.5±2.6	3.2±1.2

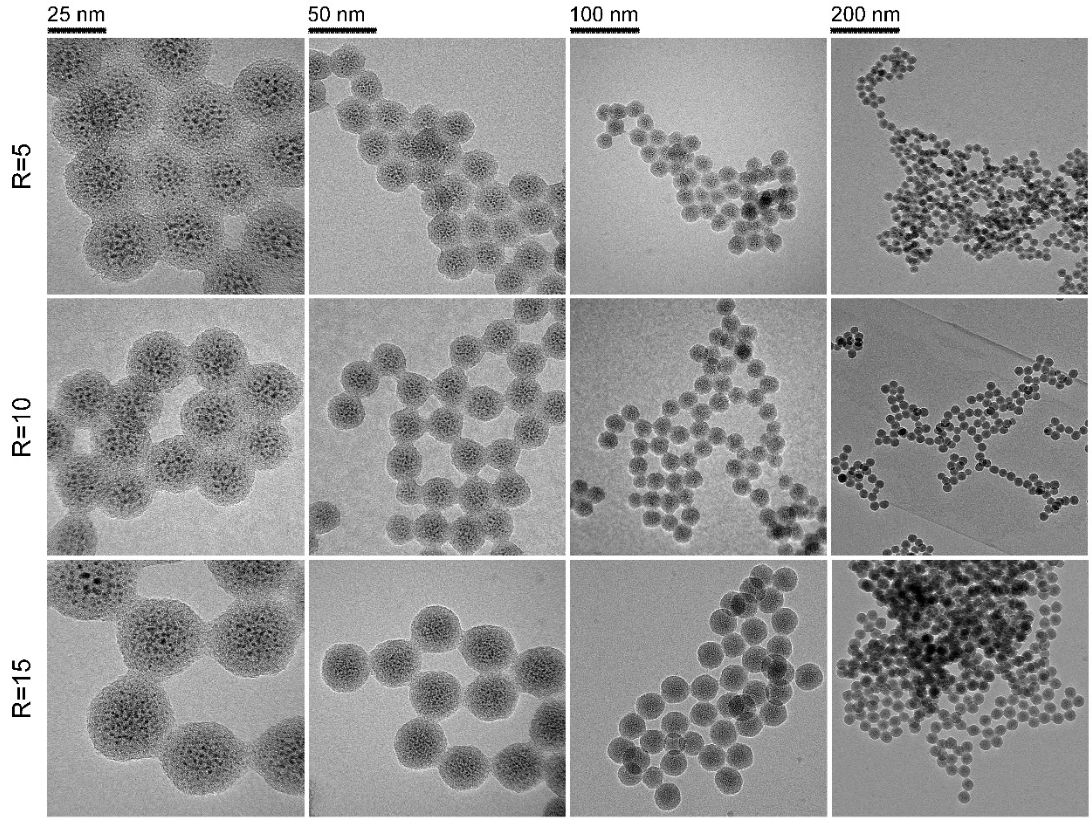

Figure S1. FTEM images of PMSN prepared using different R (R=[H2O]/[surfactant]) systems in the Igepal CO-520 system.

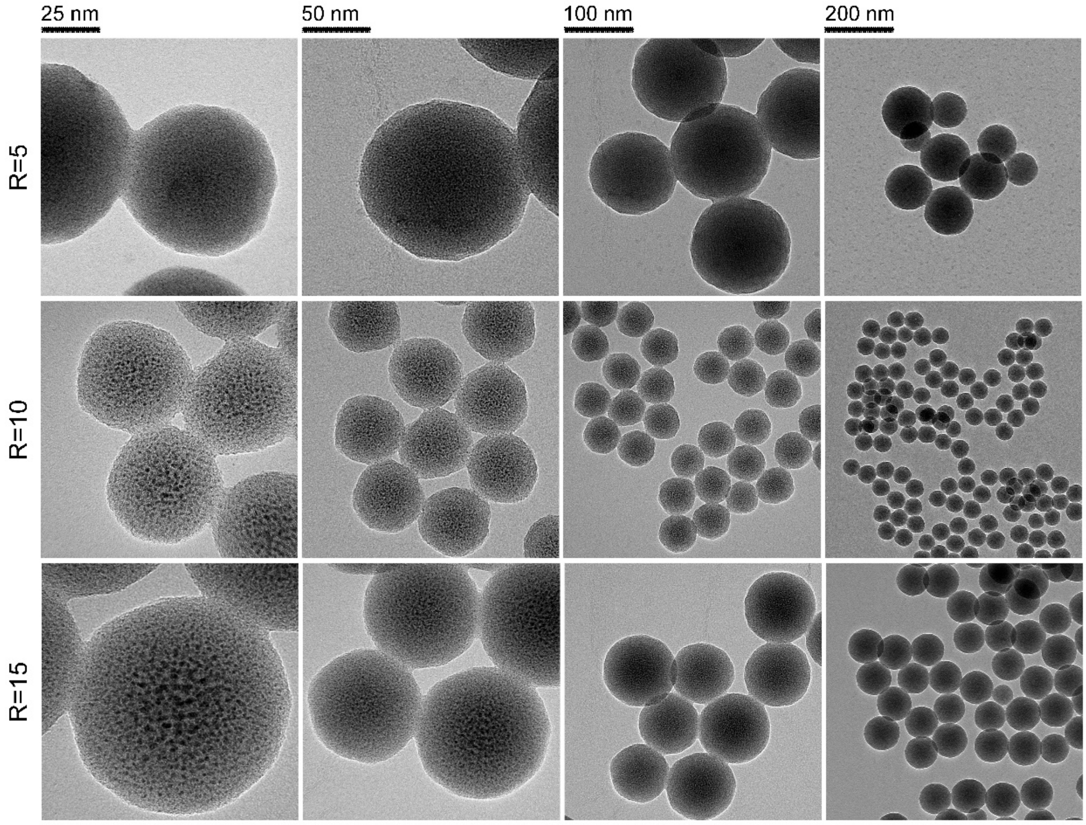

Figure S2. FTEM images of PMSN prepared using different R (R=[H2O]/[surfactant]) systems in Triton X-100 system.

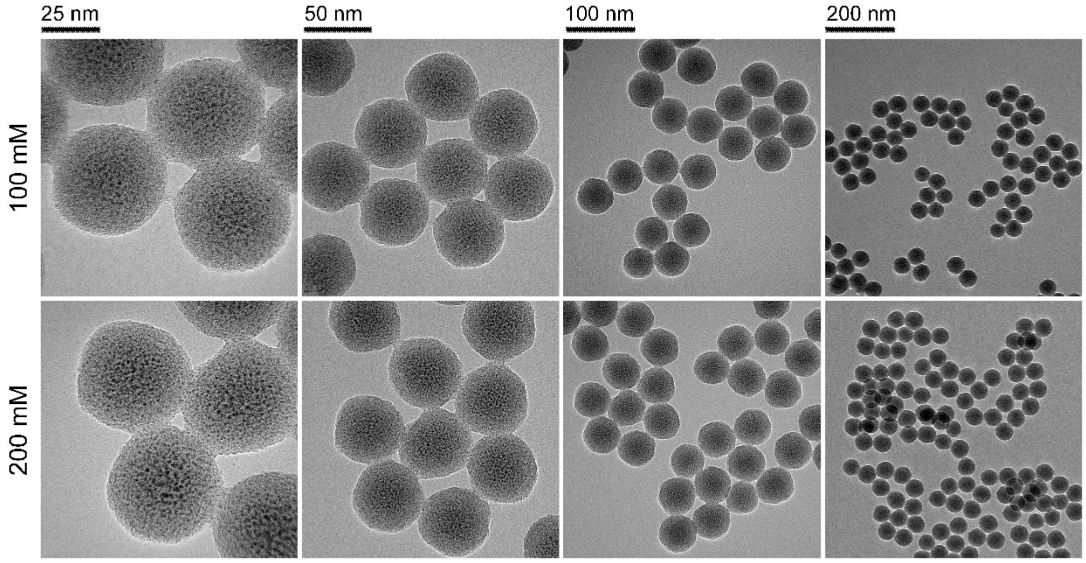

Figure S3. FTEM images of PMSN prepared using different cisplatin prodrug systems in the Triton X-100 system.

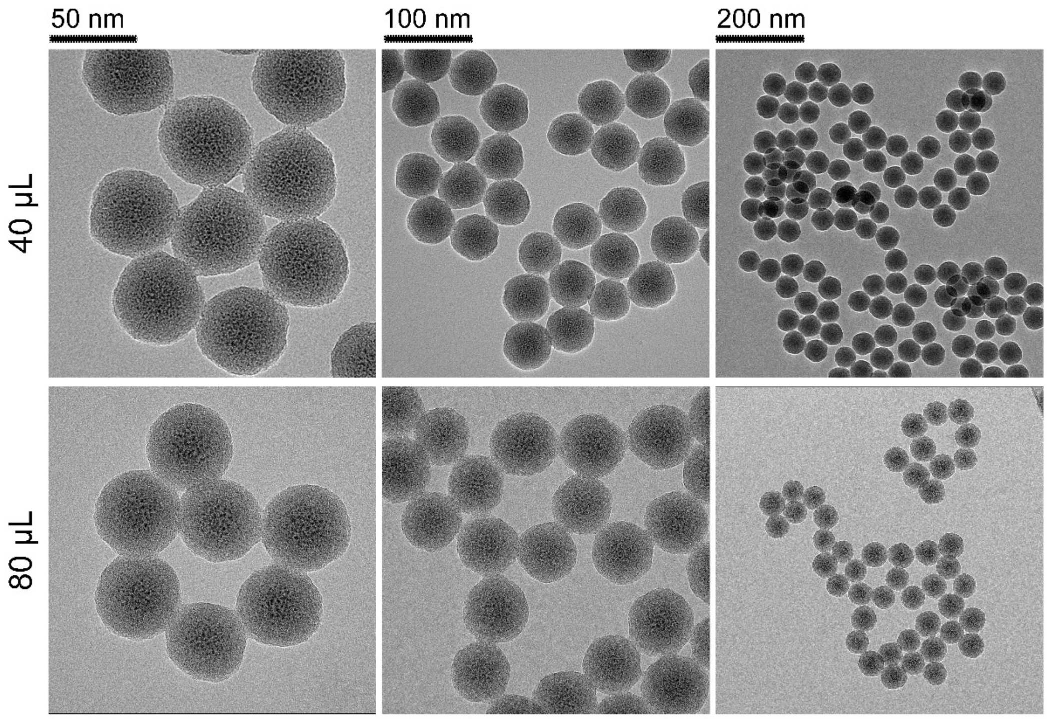

Figure S4. FTEM images of PMSN prepared using different TEOS systems in the Triton X-100 system.

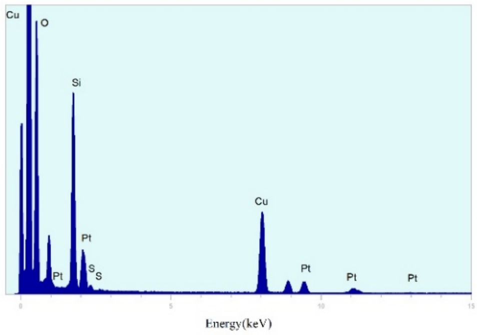

Figure S5. Energy-dispersive X-ray spectroscope (EDS) spectrum of PMON. All the major elements (Si, O, Pt, S) are found in the spectrum.

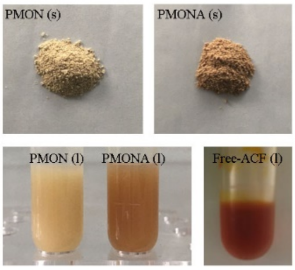

Figure S6. Digital photos of PMON and PMONA solid; PMON, PMONA and free-ACF solutions. Compared with the faint yellow color of PMON, the brown PMONA product indicates the successful loading of ACF into PMON.

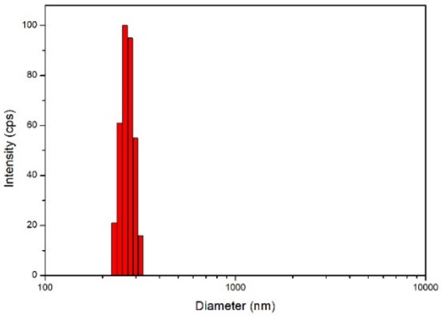

Figure S7. Dynamic light scattering (DLS) size measurement of PMONA in ddH_2O.

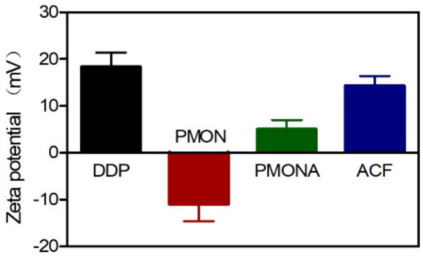

Figure S8. Zeta potential of free DDP, PMON, PMONA, and free ACF.

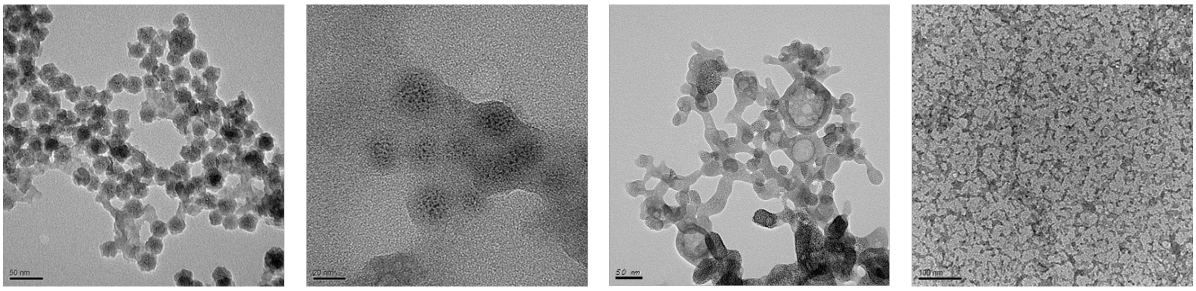

Figure S9. TEM images of PMON after 10 mM GSH incubation for 1, 2, 12, 24 hours.

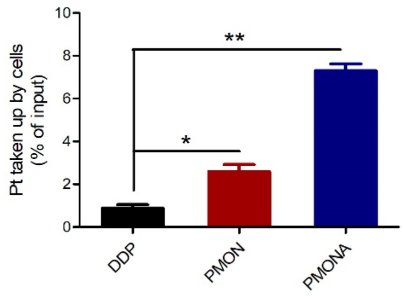

Figure S10. The uptake of Pt in A549 cells after incubation with free DDP, PMON or PMONA (5 μM cisplatin) for 18 h.

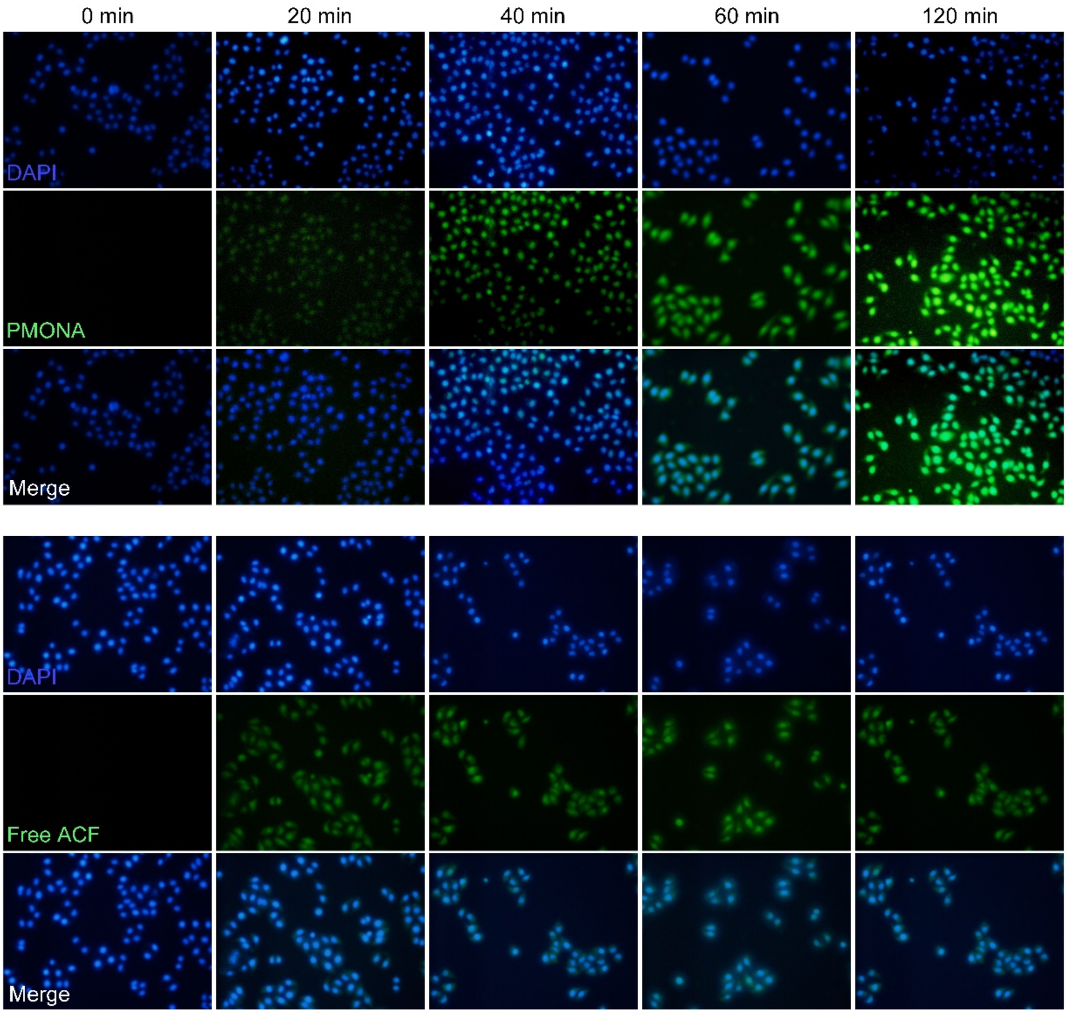

Figure S11. Intracellular location of PMONA and free ACF. A549 cells labeled with DAPI (nucleus) were treated with PMONA and free ACF (1 μM ACF) at 37°C for different periods of time and visualized under fluorescence microscope.

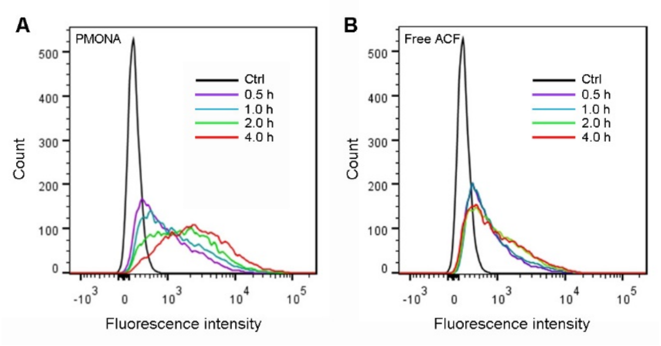

Figure S12. Flow cytometry analysis of fluorescence signals collected in A549 cells after treatment with PMONA and free ACF.

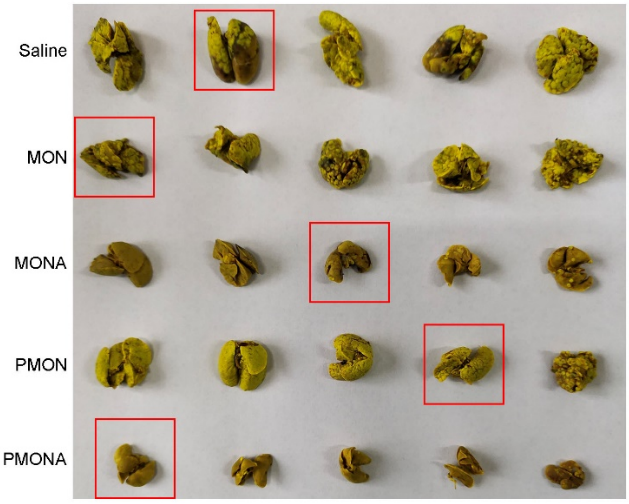

Figure S13. Optical images of lungs separated from mice receiving saline, MON, MONA, PMON and PMONA (n=5).

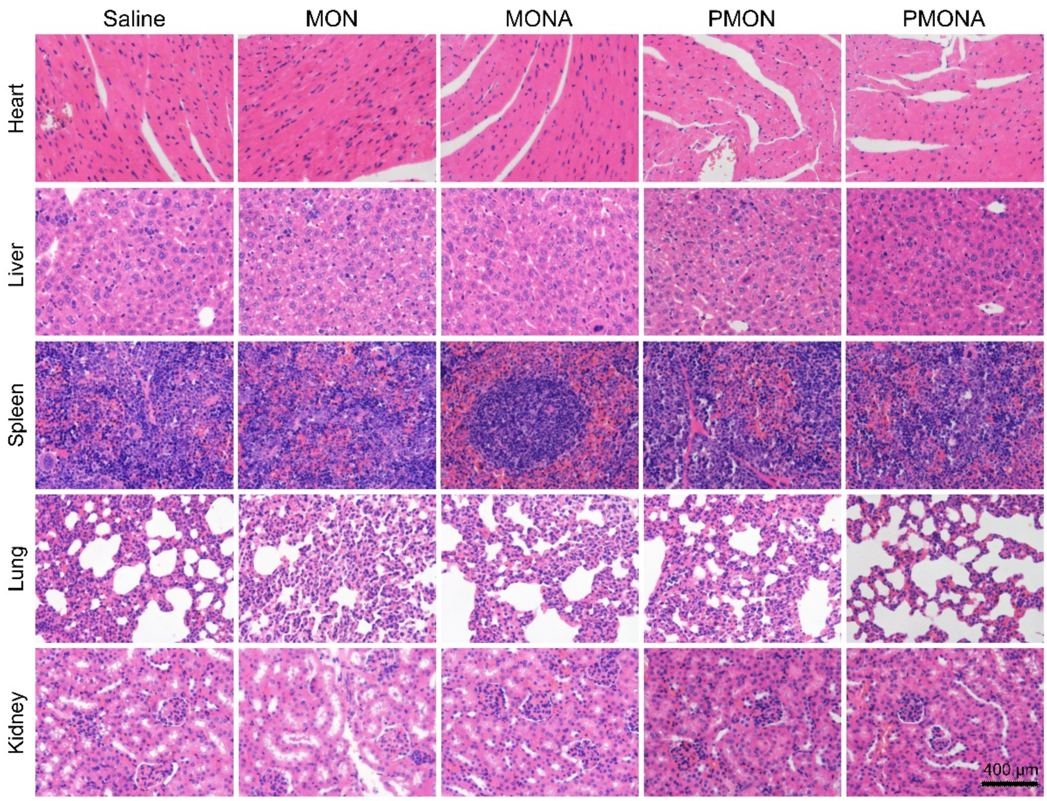

Figure S14. H&E stained image of heart, liver, spleen, lung and kidney.

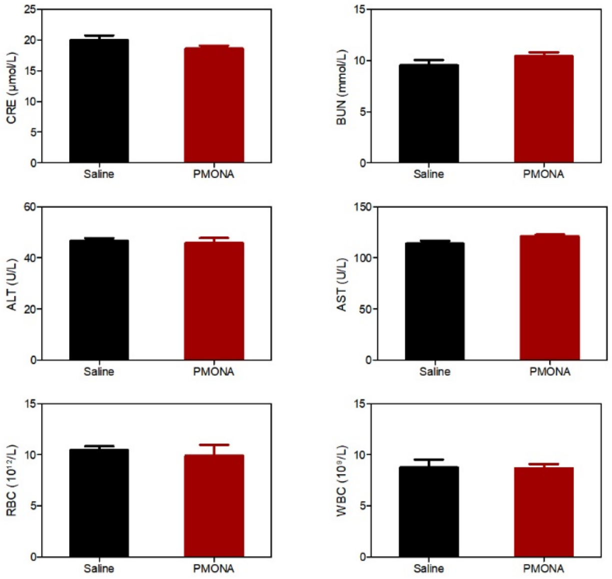

Figure S15. Blood biochemistry and hematology analysis of mice injected with saline or PMONA (day 14). CRE, creatinine; BUN, blood urea nitrogen; ALT, alanine transferase; AST, aspartate transferase; RBC, red blood cells; WBC, white blood cells.

References

(1) Zhang, X.; He, C.; Yan, R.; Chen, Y.; Zhao, P.; Li, M.; Fan, T.; Yang, T.; Lu, Y.; Luo, J.; et al. HIF-1 Dependent Reversal of Cisplatin Resistance via Anti-Oxidative Nano

Selenium for Effective Cancer Therapy. *Chem. Eng. J.* **2020**, *380*, 122540.
(2) C. He, X. Zhang, R. Yan, P. Zhao, Y. Chen, Enhancement of cisplatin efficacy by lipid–CaO_2 nanocarrier-mediated comprehensive modulation of the tumor microenvironment, Biomater. Sci. 2019.
